# Response to “Comment on ‘Effects of *in Utero* Exposure to Arsenic during the Second Half of Gestation on Reproductive End Points and Metabolic Parameters in Female CD-1 Mice’”

**DOI:** 10.1289/ehp.1511181

**Published:** 2016-03-01

**Authors:** Karina F. Rodriguez, Erica K. Ungewitter, Yasmin Crespo-Mejias, Chang Liu, Barbara Nicol, Grace E. Kissling, Humphrey Hung-Chang Yao

**Affiliations:** National Institute of Environmental Health Sciences, National Institutes of Health, U.S. Department of Health and Human Services, Research Triangle Park, North Carolina USA

In a letter in response to our paper, Williams and DeSesso questioned why a significant weight gain in arsenic (As)-exposed offspring was found in our study but not in others (Markowski et al. 2011; Markowski et al. 2012; Tokar et al. 2010; Waalkes et al. 2003; Waalkes et al. 2004; Waalkes et al. 2006). In addition, they point out that early onset of vaginal opening in response to gestational As exposure was not observed by Markowski et al. (2012) or Gandhi et al. (2012). As we mentioned in the Discussion section of our paper, some of the results under our exposure scheme do not recapitulate those observed in other studies.

The main difference that could contribute to these discrepancies is that the offspring in our study were fostered to dams that were not exposed to As during gestation. Dams exposed gestationally to As are known to produce lower-quality milk, which can result in weight deficits in their pups (Kozul-Horvath et al. 2012). In contrast, the studies mentioned by Williams and DeSesso left offspring with their As-exposed mothers. It is therefore possible that the impacts of gestational As exposure on milk quality could offset the effects of As on offspring weight gain and vaginal opening.

Regarding the lack of a dose response, our study was designed to examine the impact of two specific As doses: 10 ppb (the U.S. Environmental Protection Agency drinking water standard) and 42.5 ppm (tumor-inducing concentration). Dose–response experiments are usually performed to identify either the proper dose for further experiments or the mode of action of a particular chemical (linear, biphasic, or others). Neither of these two parameters were an end point of our study. We do not have an explanation for the different responses between the 10-ppb and 42.5-ppm treatment groups, and further studies are definitely required.

Williams and DeSesso further suggest that the control pups in our study may have been unusually small, such that our results reflect a statistical anomaly. However, data on CD-1 female weights (Lang and White 1996) indicate that the weight of our control mice at 25 weeks (approximately 34.4 g) falls in the normal range of approximately 31–42 g.

Williams and DeSesso also questioned whether an increase in body weight could contribute to the early onset of vaginal opening. This argument is indeed the focal point of our experiments, as we described in the Results and Discussion sections of the paper. Based on the analyses that examined the association between weight at weaning (postnatal day 21) and age at vaginal opening (Figure 2D of our paper), we observed that the 42.5-ppm treatment and control groups showed a positive association between weight at weaning and onset of vaginal opening. This association was not found in the 10-ppb treatment group. Although we did not have the weight records at the time of vaginal opening, we believe the population data in Figure 2D are sufficient for us to make a valid conclusion regarding the associations. The two studies mentioned by Williams and DeSesso (Markowski et al. 2012 using B6 mice; Gandhi et al. 2012 using rats) found no effect of *in utero* exposure to As on vaginal opening. Strain and species differences may contribute to these discrepancies.

In summary, in our Discussion section we fully recognize the differences between our results and those of other studies. We agree with Williams and DeSesso that the discrepancies could result from experimental conditions such as diet, strain, and species. Like all animal studies, our study provides observations on a particular strain of mouse under specific experimental conditions. The differences among studies only strengthen the point that more studies are needed to understand the mechanisms of action of As and how these different experimental conditions influence the outcomes.
